# Polysaccharide-Based Drug Delivery Systems for the Treatment of Periodontitis

**DOI:** 10.3390/molecules26092735

**Published:** 2021-05-06

**Authors:** Nicolae Baranov, Marcel Popa, Leonard Ionut Atanase, Daniela Luminita Ichim

**Affiliations:** 1 Faculty of Chemical Engineering and Protection of the Environment, “Gheorghe Asachi” Technical University, 700050 Iasi, Romania; baranov_nicolae@yahoo.com; 2 Academy of Romanian Scientists, 50085 Bucharest, Romania; 3 Faculty of Medical Dentistry, “Apollonia” University of Iasi, 700511 Iasi, Romania; danielaluminitaichim@yahoo.com

**Keywords:** periodontitis, antibacterial properties, polysaccharides, drug delivery systems, hydrogels, microparticles, nanoparticles, films, electrospun fibers, gels

## Abstract

Periodontal diseases are worldwide health problems that negatively affect the lifestyle of many people. The long-term effect of the classical treatments, including the mechanical removal of bacterial plaque, is not effective enough, causing the scientific world to find other alternatives. Polymer–drug systems, which have different forms of presentation, chosen depending on the nature of the disease, the mode of administration, the type of polymer used, etc., have become very promising. Hydrogels, for example (in the form of films, micro-/nanoparticles, implants, inserts, etc.), contain the drug included, encapsulated, or adsorbed on the surface. Biologically active compounds can also be associated directly with the polymer chains by covalent or ionic binding (polymer–drug conjugates). Not just any polymer can be used as a support for drug combination due to the constraints imposed by the fact that the system works inside the body. Biopolymers, especially polysaccharides and their derivatives and to a lesser extent proteins, are preferred for this purpose. This paper aims to review in detail the biopolymer–drug systems that have emerged in the last decade as alternatives to the classical treatment of periodontal disease.

## 1. Introduction

In the last 30 years, the field of controlled drug delivery systems, particularly the study of polymers as drug carriers, has received great interest. The polymer acts either as a support by protecting the bioactive agent during its transfer through the body until release or by its properties controlling the release kinetics. Such a controlled release system contains the bioactive principle loaded into the polymeric matrix or chemically bound (ionic, covalent) to the base chain, being administered orally, parenterally, transdermally, or surgically implanted in the body [1]. The drug will then be released at the site of the disease by diffusion, by hydrolysis of the chemical bonds between it and the support, or by erosion of the surface of the polymeric matrix [2].

The polymeric matrix must have an optimal combination of binding properties for compounds that work in or in contact with the human body, which makes biopolymers (proteins, polysaccharides) ideal candidates. Although the mechanisms of action of the release systems thus obtained are not yet fully elucidated and their technologies are not yet optimized, the results of some clinical experiments attest to the special value of some natural macromolecular compounds, and to a lesser extent of synthetic polymers, as adjuvants for the conditioning and immobilization of biologically active substances.

The main constraints that a polymer for bio-applications must meet are the (i) biocompatible and biodegradable. This assumes that the polymer must degrade in vivo into compounds that are easily eliminated from the body. Otherwise, the polymeric system must be surgically removed after completely or almost completely releasing the drug. (ii) The degradation products must be non-toxic and not create an inflammatory response in the body. (iii) Degradation of the polymer must occur within a reasonable period of time. In addition to these constraints, the choice of polymeric matrix also takes into account several criteria: (i) Molecular weight and polydispersity of molar masses. (ii) Basic chain geometry: linearity or cyclicity, branching, reticulation, etc. (iii) The chemical nature of the polymer, such as its chemical composition, aqueous solubility, and ionic charge. (iv) The polymer–drug relationship, such as the type of chemical bond that the partners can form (covalent, ionic, or coordinative); the physical interactions between partners, if no chemical bonds are involved; location of the pharmacologically active agent in the polymer (per chain, as a substituent, or included in the matrix). (v) The stereochemical phenomena. 

The mentioned restrictions are fulfilled by few synthetic polymers, but mostly by biopolymers. Of this large category, the most used are polysaccharides and proteins; this review focuses only on polysaccharides and their derivatives. Given the specific diseases of the oral cavity, among which periodontal disease ranks first, the classes of drugs associated with different types of polysaccharide-based supports are those with anti-inflammatory and antimicrobial action on the microbial flora that causes periodontitis.

Numerous forms of the presentation of polymer–drug systems are currently known, these being chosen depending on the nature of the disease, the administration mode, the type of polymer used, etc. One of them is hydrogels (in the form of films, micro-/nanoparticles, implants, etc.), in which the drug can be included, encapsulated, or adsorbed on the surface. Another possibility is the direct association of the biologically active compound with the polymer chains by covalent or ionic binding (polymer–drug conjugates).

The literature abounds with a huge amount of articles dealing with the problem of classifying polymer–drug systems according to the way partners associate, how they are formulated, how they respond to external stimuli, the nature of the support polymer, the mechanism and kinetics of the release process, etc. This review will not refer to these aspects but will only investigate the polymer–drug systems with applications in the treatment of periodontitis. Moreover, the commercial applications/clinical trials are out of the scope of this review.

**Figure 1:**
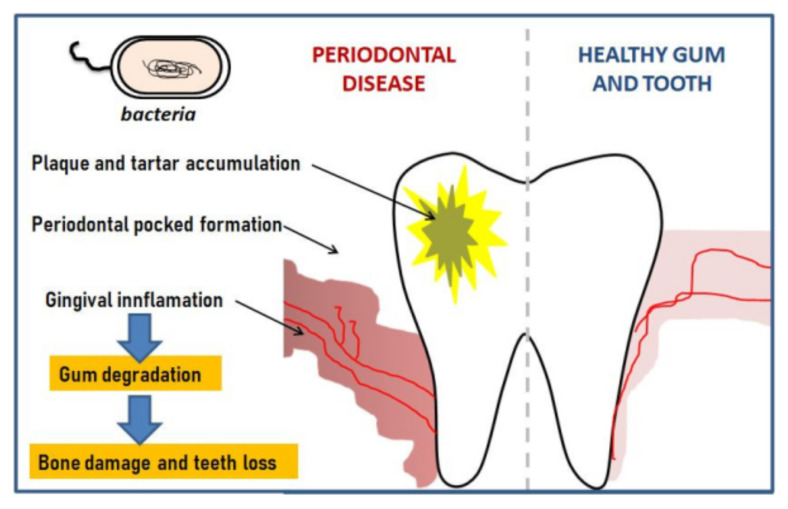
Schematic illustration of the stages of evolution of periodontal disease. Reprinted from ref. [4].

Apart from trauma produced by injuries, the most common diseases of the oral cavity are tooth decay, periodontal disease, oral infections, and cancer. It is estimated, for example, that approximately 48% of the US adult population is affected by periodontitis, but similar results have been reported in other countries. Periodontitis is therefore a chronic inflammatory disease of the periodontal tissue caused by pathogenic microorganisms and characterized by the destruction of the supporting structures of the teeth [3]. The inflammation is first located in the gum but then penetrates deep and in the absence of treatment creates bags colonized by anaerobic bacteria that erode the supporting ligaments of the tooth until it is lost; the progress of the inflamed bags can take several years. Several inflammatory and degenerative stages of the tissue surrounding the tooth are highlighted, the gum, periodontal ligaments, enamel, and alveolar bone being affected in turn; these stages in the evolution of the disease are suggestively presented in Figure 1 [4].

The understanding of the etiology and pathogenesis of periodontal disease has made significant progress in recent decades. In all forms of the disease, the strength of the microbial attack depends on the virulence of the microorganisms, the amount and composition of the bacterial plaque, their ability to invade the tissues, and their metabolic products [5]. The periodontal microbiota is a complex community of microorganisms, many of which are still difficult to isolate in the laboratory [6,7,8,9].

Many species function as pathogens, and those that function as pathogens in a site may exist in small amounts and in healthy sites. Bacterial accumulations cause vascular changes typical of inflammatory reactions, the result of a loss of vascular fluid and the migration of polymorphonuclear leukocytes from blood vessels into tissues and gingival sulcus. There are also early losses of apical collagen from the junction epithelium that begin during the time the bacterial plaque is deposited. The stage of chronic gingivitis is installed but limited to the tissues located very close to the sulcular and junctional epithelium. The supragingival plaque, if left to grow normally, accumulates and will cause gingival inflammation with the appearance of tissue products that serve as nutrients for Gram-negative anaerobes. At the same time, inflammation together with bacterial enzymes open a gateway to the tissue of high molecular weight products from bacteria that penetrate tissues, ultimately causing periodontal disease.

Among the bacteria that activate the destructive processes of the host tissues, the most important are: Porphyromonas gingivalis, some types of Prevotella intermedia, Tannerella forsythia, Aggregatibacter actinomycetemcomitans, Fusobacterium nucleatum, etc. [5]. 

Periodontal disease refers to all pathological manifestations with a multifactorial etiology, which appear as a consequence of interactions between microbial factors (determinants) and those of the host but also possible environmental influences (favorable factors) that affect the supporting periodontium and lead to tissue destruction, the experience of chronic evolution (with periods of activity and inactivity), and the requirement of a complex and long-term treatment [10,11]. It is characterized by gingival inflammation, the formation of periodontal pockets, and the destruction of the periodontal ligament and alveolar bone, as well as by the mobilization of teeth [12].

In fact, the prevention of periodontal disease is very important, and the best way is to remove its causes, which is the formation of bacterial plaque. Treatment often involves the systemic administration of the free drug, with the effectiveness of the treatment being reduced as the concentration of the active principle decreases rapidly over time to the sub-therapeutic level, when a new dose is required. The trend of recent years in the treatment of this disease has shifted to the local release of antibiotics, antimicrobials, and anti-inflammatory drugs, and this is through the local administration of drug delivery systems. An ideal system of this type consists of a biodegradable, bioadhesive support, based on natural and rarely synthetic polymers, capable of releasing the active principle relatively controlled and for as long as possible. Several types of such systems were investigated to date, such as electrospun fibers, tapes, inserts/implants, films, gels, hydrogels, polymer–drug conjugates, dendrimers, micelles, nanocapsules, micro- and nano-spheres, and particularly micro-/nano-capsules and liposomes.

Local administration has a number of apparent advantages over the systemic one, directly targeting the affected area, but even in this case repeated doses of the active ingredient are required to maintain a relatively constant concentration of the active ingredient, minimizing systemic side effects [13,14]. The most common formulations for local use in buccal cavity are semi-solid or liquid, especially because they are both easy to administer and borne by the patient. However, the disadvantage is the poor retention in the oral cavity, and, therefore, the achievement of a therapeutic effect is far below the optimal one. As a result, ways have been sought to solve these disadvantages, one of which is the association with polymers [15]. The use of a local system of release of the drug when placed in the periodontal pocket allows—and, therefore, maintains—a high and relatively constant concentration in time of the drug, which is more difficult to remove by salivary secretion and, therefore, cannot be swallowed by the patient, as well as preventing unwanted side effects that the free drug can generate [16].

## 2. Polymer–Drug Systems

An ideal mechanism for the release of drugs from polymer matrices should be characterized by zero-order kinetics (constant velocity) or at most of the first order. 

Depending on the formulation and the application method, the duration of the drug release can vary from a few hours to a few days, months, or even years (in the case of implants). While the advantages of using such systems are significant, the potential disadvantages cannot be ignored: possible toxicity of the materials used; unwanted degradation of the products formed; surgical action required to implant and/or to remove the implant; the possibility that the system causes patient discomfort; and the cost of preparing and processing controlled release systems, which can be considerably higher compared to traditional pharmaceutical forms.

In the following, a detailed review of the different types of drug delivery systems based on polysaccharides and their derivatives and anti-inflammatory, antibacterial, etc. drugs, with potential application in the treatment of periodontitis that have been reported to date in the literature are presented.

### 2.1. Gels

Gels are nonfluid colloidal networks or polymer networks that are expanded throughout their whole volume by a fluid [17]. The network may be covalent in nature, formed by cross-linking the linear chains of a polymer, by nonlinear polymerization, or formed through the physical aggregation of polymer chains, caused by ionic bonds, coordinating bonds, hydrogen bonds, production of coils, hydrogen bonds or hydrophobic interactions, crystallization, or helix formation, that results in regions of local order acting as the network junction points. If the regions of local order are thermally reversible, the resulting swollen network may be termed a “thermoreversible gel”. A special category of gels is the hydrogels, which will be discussed in the Section 2.2.

Due to the nature of the constituent polymers, the gels that can be administered in the oral cavity are mucoadhesive systems [18], which are easy to prepare. They are applied sublingually or in the periodontal pocket using a cannula or syringe [19] but have the disadvantage of a quick release of the active ingredient. Gelling occurs instantly through various mechanisms, which involve chemical or physical factors. For example, when using ionic polymers, the presence of polyions of the opposite charge to the ionic polymer used can cause gelling. The process occurs in situ when the polymer solution is instilled (polyanionite, for example) in the periodontal pocket, under the action of electrolytes, such as Ca^2+^, Mg^2+^, and Na^+^, which are inevitably found in the fluids of the oral cavity and cause a change of the ionic strength of the polymer solution. Polymers that can be used to form gels in situ through this mechanism include alginates, HA, and GG. The gelling speed is determined by the osmotic gradient around the gel surface and the change in ionic strength when instilling the solution produces gelling. 

#### 2.1.1. Chitosan-Based Gels

Chitosan (CS) fulfills all of the aforementioned constraints for polymers usable as drug carriers, to which its strong mucoadhesive character and its intrinsic antimicrobial activity is added. CS-based gels, starting from solutions of different concentrations, are able to include and release tetracycline hydrochloride and metronidazole benzoate. The optimal concentration of the CS solution has been shown to be 3%, which allows the modulation of the dose of the drug substance in an optimal way for the local therapy of periodontitis [20]. Atorvastatin is an anti-inflammatory drug commonly used to treat inflammation in the oral cavity, but its limited solubility in water makes it less bioavailable. CS-based gels loaded with various anti-inflammatory drugs, including atorvastatin, have recently been reported by Özdoğan et al. In presence of atorvastatin, the bioadhesive property of the formulations was found to increase, which would retain the delivery system at the application site and maintain drug levels at the desired amount for a longer period of time [21]. The results showed that combining the drug with CS improves and enhanced the anti-inflammatory effect of atorvastatin as well as bone and tissue healing in vivo in periodontitis-induced rats [22]. In order to increase the bioavailability of the drug, the authors have prepared solid dispersions of the drug in some polymers (PEG, Pluronic F-68, CS) by using different drug/polymer ratios. CS gels incorporated with particles of atorvastatin exhibited suitable viscosity and a bioadhesive property for a high retention time of the drug at the application site. These atorvastatin-loaded formulations exert anti-inflammatory activity enhanced by the presence of CS [23]. 

Anti-inflammatory and antimicrobial CS-based nanogels using triclosan and flurbiprofen were obtained. Flurbiprofen was directly loaded into the nanogel, and Triclosan was prepared as nanoparticles using poly(ε-caprolactone) (PCL) [24]. The two systems showed a sustained and rapid in vitro release of drugs. The in vivo tests on rats proved an improved anti-inflammatory action of gels and reduction of bacterial plaque.

CS gels loaded with *Schinus molle L.* essential oil were evaluated for activity against bacteria associated with periodontal disease in dogs [25]. These gels showed their potential as a delivery system for *Schinus molle L.* and presented ideal physicochemical and rheological properties required for application on buccal tissue, such as good stability and antimicrobial activity against Gram-positive and Gram-negative bacteria (*Staphylococcus* spp., *Streptococcus, Corynebacterium* spp., *Pseudomonas* spp.) associated with canine periodontitis.

Thermoresponsive gels are attractive; they are liquid at low temperature and thus can be easily injected into deep pockets, and then they can form gels under body temperature. In the formulations reported so far, CS is associated with Poloxamer, a triblock copolymer with a hydrophobic central segment. Thermosensitive and mucoadhesive polymer-based sustained release moxifloxacin in situ gels for the treatment of periodontal diseases were reported recently by Shashala et al., [26]. The gel formulation consists of a mixture of Poloxamer and CS, which is formed during the injection in the periodontal bag at a temperature of 37 °C. The mucoadhesive nature of the polymers aids the gel to adhere to mucosa in the periodontal pocket for a prolonged time and releases the drug in a sustained manner. In vitro drug release studies demonstrated a sustained release for 8 h, and the antimicrobial studies proved a promising antimicrobial efficiency against *Aggregatibacter actinomycetemcomitans* and *Streptococcus mutans.* A combination of CS and Poloxamer 407 generates a heat-sensitive mucoadhesive gel that releases levofloxacin and metronidazole for 48 h [27,28]. 

#### 2.1.2. Gels Based on Miscellaneous Polysaccharides

Microbial polysaccharides (gellan) or cellulosic derivatives have high solubility in water, the ability to form gels at higher concentrations, bioadhesive properties, and are being used especially in mixtures with other polysaccharides or synthetic polymers.

Formulations containing hydroxypropyl methylcellulose (HPMC), CMCNa, Carbopol 940, and propolis extract were reported. The antibacterial activity of the most promising formulation against *Porphyromonas gingivalis* was investigated by the disk diffusion method and it appeared that it was efficient in the treatment of periodontitis, which recommends it for clinical evaluation [29]. Doxycycline and metronidazole were incorporated recently into a hydroxyethylcellulose (HEC) poly(vinylpyrrolidone) (PVP)/calcium polycarbophil gel [30]. The antimicrobial activity was tested against *A. actinomycetemcomitans*, *S. sanguinis*, *P. micra*, and *E. corrodens*, and it was noticed to have an inhibitory effect during the first 24 h, which was kept constant until the 13th day. The in vivo test suggests that the formulated gel is effective against bacteria that are already present or will colonize the periodontal pocket.

This category of polysaccharides, especially in mixtures with Poloxamer, can generate heat-sensitive gels capable of sustained and constant release of the included drug. A mixture of Poloxamer 407 and methylcellulose generates a thermosensitive gel at room temperature, releasing simvastatin in a controlled manner for 10 days [31]. 

Recently, a gel based on Poloxamer 407, Carbopol 934 P, and gellan gum (thermo- and ion-sensitive), which is formed in situ, was prepared for the local release of moxifloxacin hydrochloride in periodontal pockets, showing a high in vitro antibacterial activity against *Staphylococcus aureus* and *Escherichia coli*, with prospects for clinical trials [32]. 

### 2.2. Hydrogels

Hydrogels are a derived form of gels, representing a three-dimensional solid resulting from the hydrophilic polymer chains being held together by cross-linking. The cross-linking methods that bond the polymers of a hydrogel fall under two general categories: physical and chemical. Hydrogels are networks capable of absorbing large amounts of water (over 90%, sometimes up to over 10,000% in the case of superabsorbent hydrogels) with the network remaining intact, unlike gels that can be disintegrated and fluidized by dilution. Being able to include high amounts of water as well as substances dissolved in it, hydrogels often possess physicochemical properties close to those of the native extracellular matrix [33,34].

Given the presence of functional groups, such as amines, carboxylic, and hydroxyl, in their structure, hydrogels can respond to various physical or chemical stimuli and are considered in many cases as “smart materials”. Owing to this feature, they can encapsulate and especially release various chemical compounds, including drugs. A recent review discusses the “smart” hydrogels, which are able to respond to various external stimuli, such as temperature, pH, light, glucose, enzymes, pressure, magnetic field, electric field, or ultrasound, with the potential to be used in the treatment of periodontal disease [35]. Table 1 summarizes the latest information in the literature on obtaining hydrogels based on polysaccharides for inclusion in drugs, with applications in the treatment of periodontal disease, the table is organized by the cross-linking method and the nature of the polysaccharide. The main characteristics of the hydrogel and the effects on periodontal disease are presented.

#### 2.2.1. Physical Hydrogels

It is well known that these networks are made by the interaction of the polysaccharide polyanion with at least divalent ions of opposite charge.

CS is by far the most widely used polysaccharide for obtaining hydrogels. CS derivatives or mixtures thereof with other biocompatible polymers, particularly with mineral powders (hydroxyapatite), are also reported in the recent literature as being able to generate hydrogels usable not only for the loading/releasing of anti-inflammatory or antimicrobial drugs with applications in the treatment of periodontitis but also for alveolar tissue regeneration. The presence of amino groups that are quaternized in an acidic environment makes the ionic interaction with polyvalent anions possible (sulfate anion, polyphosphate, etc.).

Recently, CS and CS derivative-based thermosensitive hydrogels have gained great attention. They are formed in situ, at a physiological temperature, when the liquid mixture of its components is introduced into the periodontal pocket.

Representative of these are hydrogels based on CS and cross-linked derivatives with β-glycerophosphat discodic salt and rapid formation, which are capable of releasing the bioactive agent (anti-inflammatory, antibacterial, antibiotic) in fluids that simulate saliva depending on its nature (after 18 h or even a month [41]); the advantages over the classical, periodic administration being obvious. Hydrogel administration is done by direct injection into the periodontal pocket [40] and, in addition to the anti-inflammatory and antibacterial action, such systems can exhibit a positive effect on the endogenous repair of alveolar bone [52] and can be used for periodontal treatment in an experimental periodontitis model, such as ligature-induced periodontitis [37,38].

Microbial polysaccharides such as gellan or curdlan can generate hydrogels by ionic gelling, in the presence of bivalent metal ions (Ca^2+^, for example). Gellan and nano-hydroxyapatite-based biocomposites loaded with chlorhexidine (50 µg/m) and bone marrow mesenchymal stem inhibit *Enterococcus faecalis* in a concentration-dependent manner and are recommended for treating infectious bone defects caused by refractory periradicular periodontitis [42]. A hydrogel based on curdlan and polydopamine loaded with clorhexcidine acetate is a potential candidate for periodontal antibacterial treatment by combining photothermal and antimicrobial effects simultaneously [43].

#### 2.2.2. Chemical Hydrogels

Chemical cross-linking exploits the presence of functional groups of the polysaccharide -OH, -NH_2_, and -COOH in reaction with bifunctional compounds with complementary functions (dialdehydes, epichlorohydrin) to create the three-dimensional structure. Another way consists of the functionalization of the polysaccharide with polymerizable groups, so that by chemically, thermally, or photochemically initiated polymerization, hydrogel networks are obtained.

Although considered toxic, glutaraldehyde (GA) can be used, in small amounts, as a chemical cross-linker. For example, a hydrogel based on CS cross-linked with AG, loaded with doxycycline showing a release of 40 µg/mL after 24 h can be used for periodontal regeneration [44]. Interestingly, a local drug carrier for diabetics’ periodontitis therapy is a glucose-sensitive CS-poly(ethylene oxide) (PEO) hydrogel of a semi-interpenetrated type, which can release metronidazole at a higher glucose concentration and has a great capacity to inhibit *Porphyromonas gingivalis* [46]. Using the same cross-linking agent, mesoporous hydroxyapatite/CS biocomposite hydrogels loaded with recombinant human amelogenin were obtained; they can inhibit the growth of periodontal pathogens and promote the formation of bone and cementum-like tissue [47].

Cellulose and its derivatives can be cross-linked with epichlorohydrin, biocomposites containing cellulose nanofibers, and κ-carrageenan oligosaccharide. The biocomposites loaded with surfactin and herbmedotcin exhibit antibacterial activity against *Streptococcus mutans*, *Porphyromonas gingivalis*, *Fusobacterium nucleatum*, and *Pseudomonas aeruginosa* [48]. Xyloglucan from tamarind seeds was used as a cross-linker to obtain an injectable and mucoadhesive methyl cellulose-based hydrogel, obtained directly in the periodontal pocket for the in situ release of metronidazole [48].

Interestingly, hydrogels obtained by in situ cross-linking through different modes of activation have been reported relatively recently by different groups of researchers. Light curing is one of the methods used for this purpose. The polysaccharide is pre-functionalized by introducing unsaturated groups as a substituent on the base chain. A hydrogel based on CS-methacrylate loaded with metronidazole containing glucose oxidase immobilized on its surface is able to rapidly and correspondingly adjust its inner pore structure to control the loaded metronidazole release film with the rising glucose concentration, so it is an important candidate for diabetics’ periodontitis therapy [49]. Another variant is the use of glycidyl methacrylate as a cross-linker, which in the presence of CS and carboxymethyl CS generates a hydrogel by photopolymerization, whose gelling time decreases with the photoinitiator concentration [53].

#### 2.2.3. Double Cross-Linked Hydrogels

Double cross-linking can be a method of obtaining hydrogels, which is applied in order to partially replace the covalent cross-linker with an ionic cross-linker, and thus, to reduce the toxicity of the obtained product. However, a minimum amount of covalent cross-linker must be maintained to ensure the required structural and mechanical stability of the hydrogel.

**Figure 2:**
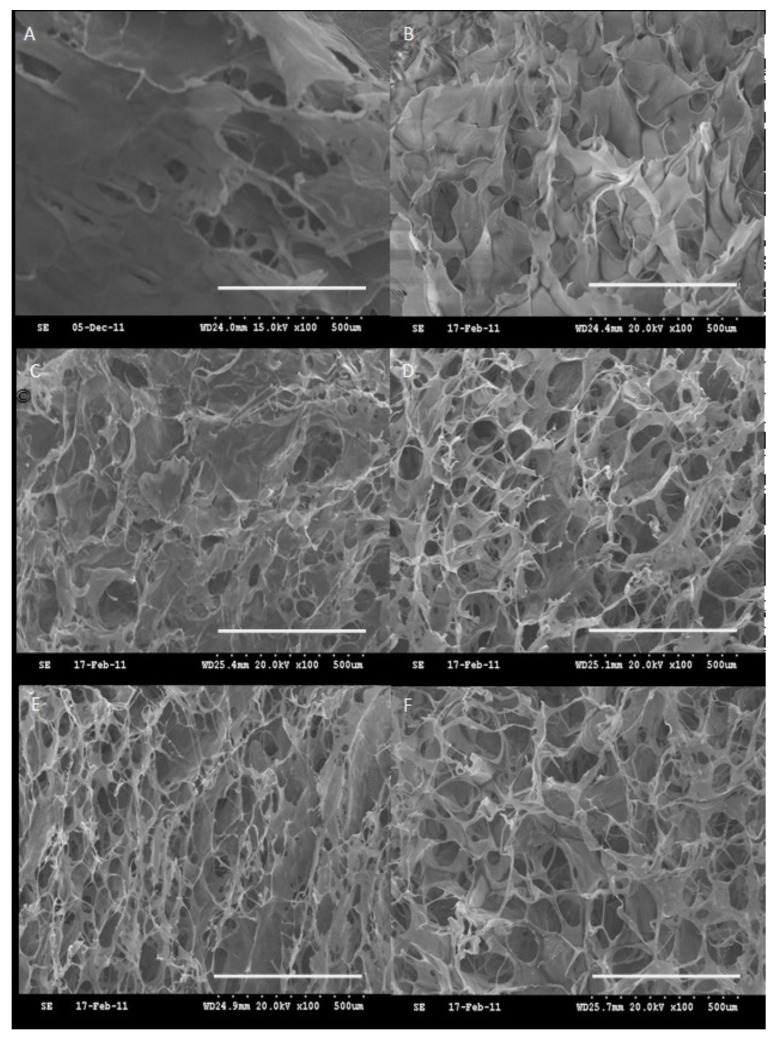
SEM morphology (scale bar = 500 μm) of (**A**) lyophilized collagen and oxi-HA/collagen hydrogel with the connective pores clearly shown in (**B**) CH–10%; (**C**) CH–20%; (**D**) CH–30%; (**E**) CH–35%; and (**F**) CH–40% (percentages indicate the concentration of the polymer mixture in the solution from which the hydrogel was obtained after cross-linking and lyophilization). Reprinted from ref. [50].

Wei et al. prepared a biocomposite based on collagen cross-linked with oxidized HA and with oligomeric proanthocyanidins, integrated with tricalcium phosphate [50], with the intention to include tetracycline for the local release of the drug into the periodontal pocket. The obtained hydrogels show a macroporous morphology with interconnected pores, whose diameter varies between 50 and 250 μm (Figure 2). The biocomposite exhibits a good mechanical strength, as well as a good biocompatibility in promoting the proliferation of MG-63 cells. The release of the antimicrobial drug is produced with a “burst effect” (50% released drug) after 24 h and continues with an effectiveness of 93% after 5 days.

An interesting injectable hydrogel consisting of a PVA matrix that included CS-decorated metronidazole microcapsules was obtained by dual ionic and covalent cross-linking, with the network being created by 4-carboxyphenylboronic acid bridges [51]. The hydrogel is bioadhesive, injectable, and administered directly in the periodontal pocket where it exerts an antibacterial effect for 14 days according to in vitro tests and for a week according to in vivo tests on a rat model of periodontitis.

The summary examples presented are important findings in favor of the use of injectable hydrogels, especially of those sensitive to external factors, which can be formed by cross-linking in situ, e.g., directly in the periodontal pocket.

### 2.3. Films

The films are matrix systems similar to nanofibers and bands, with the drug being dispersed throughout their mass and the release being achieved predominantly by diffusion but also by erosion or even dissolution of the matrix. Their mucoadhesive behavior in most cases, as well as the flexibility and ease of preparation, make them preferable compared to other formulations. In addition, buccal films can protect the wound surface, thus reducing aches and treating the oral diseases more effectively. The size and shape of the film can be easily shaped so that it fits perfectly into the periodontal pocket into which it is inserted. Larger films can be applied even to the mucous membrane of the cheek but can also be cut into smaller pieces that can be placed directly in the periodontal pocket [54].

The mucoadhesive character of CS, to which its intrinsic antibacterial activity is added, determines its preferential use in making oral films. Ganjoo et al. obtained medium molecular weight CS-based films and loaded them with lincomycin hydrochloride and inserted them directly into the periodontal pouch, using solvent casting technology [55]. Although there are no strong chemical or physical interactions between the polymer matrix and the drug, its release occurs without a burst effect, practically linear up to 100 h when total release does not yet occur.

CS-based films charged with cyclohexidine have been shown to be effective against *Porphyromonas gingivalis*, the efficacy being higher even compared to free cyclohexidine [56]. The same polymer generates biodegradable films in which metronidazole and levifloxacin can be loaded and ensures a slow and steady release over time [57]. Combining the antimicrobial character of CS with the ability to load drugs with an antibacterial action, films were obtained based on this polysaccharide or in combination with HPMC, MC, HEC, or PVA loaded with a cetylpyridinium active principle, which has a bactericidal activity against some Gram-positive bacteria such as *Streptococcus mutans* and even against some Gram-negative bacteria in higher concentrations [58]. The films with PVA showed an antimicrobial activity comparable to Cetylpyridiunium chloride, but it is preferable due to the longer residence time in the periodontal pocket that ensures a longer action on *Streptococcus mutans*.

Labib and co-workers obtained films based on CS, HPMC, and Carbopol 934 loaded with pentoxifylline and metronidazole, which proved to be advantageous in clinical trials [59]. 

CS- and PVP-based films were optimized in terms of composition to obtain a high swelling capacity in aqueous media and mechanical properties suitable for use as release systems in the oral cavity [60]. The main goal was to optimize the oral mucoadhesive properties but also the loading capacity of tenoxicam. The in vivo salivary pharmacokinetic study of the optimized film revealed rapid and sustained salivary tenoxicam delivery in the buccal cavity, so we can conclude that the polymer–drug system can be an alternative to oral therapy for the treatment of chronic periodontitis and can provide a means to overcome the off-targeted side effects of an orally delivered drug.

Layered films based on CS and PCL, both polymers being biodegradable, were loaded with metronidazole, proving good mechanical properties and a slow release of the drug [61]. 

Monoadhesive films loaded with biologically active compounds with action against bacterial plaque have been reported using other types of polysaccharides. For example, ciprofloxacin-loaded buccal films were prepared using mucoadhesive polymers as sodium cellulose and sodium alginate, using the solvent evaporation method [62]. The films showed good physicochemical properties, mucoadhesion, high drug loading capacity, and high ex vivo drug release after 12 h. The significant reduction in the bacterial count in a periodontitis model proved a potent anti-periodontitis activity. Another study reports the preparation and characterization of films based on PCL and alginic acid, loaded with metronidazole and doxycycline [63]. The mechanical properties (tensile strength) are affected by the presence of the drug, reducing with its concentration. On the contrary, water absorption capacity increased with the increase of the drug content. The film can be applied in the form of a ring around the tooth that is to be extracted after a week. The application in the form of a disposable toothpick allows the dentist to access untouched areas of deep pockets and fistulas after mechanical debridement.

Multilayer films made by the solvent evaporation technique were obtained using surface erodible polymers, particularly cellulose acetate phthalate (CAP) and Pluronic F-127. They were loaded with metronidazole, ketoprofen, doxycycline, or simvastine [64]. This bio-erodible system releases the drug sequentially over time, depending on the pathogenesis of the disease, acting on its various stages and thus ensuring a more appropriate treatment. Another multiple layer film was developed by double casting followed by a compression method starting from CMCNa (which constitutes the core layer), sodium alginate (intermediate layer that loads the drug), and thiolated sodium alginate, which constitutes the outer drug free layers to enhance mucoadhesion and allow sustained release of the drug—metformin hydrochloride—for 12 h. Clinical results indicated improvement of all clinical parameters six months post treatment. The results suggested that local application of the mucoadhesive multiple layer films loaded with metformin hydrochloride showed great potential in the nonsurgical management of moderate chronic periodontitis [65]. 

Alginate in combination with bacterial cellulose and gelatin can generate multifunctional composites capable of sustainably loading and releasing curcumin. [66]. Hydrated films adhered firmly onto the skin, tests were performed on pig skin in artificial saliva, revealing an adhesion time of up to 6 h. Curcumin-bearing films had substantial antibacterial activity against *Escherichia coli* and *Staphylococcus aureus,* which are not cytotoxic to human keratinocytes and human gingival fibroblasts but exhibited potent anticancer activity in oral cancer cells. The versatile character of the films allows the achievement of the desired characteristics depending on the application, some of them being the realization of patches for skin care or the treatment of periodontitis and cancer of the oral cavity. Films based on alginate and gelatin incorporating nanoparticles of hydroxyapatite and an antibiotic were prepared for treating infrabony periodontal defects. It was found that there is the possibility of tailoring drug release (of tetracycline) from the nanocomposite film by varying the drug loading method; the release period can be extended up to 10 days [67]. 

HPMC-based bucoadhesive buccal films for local release of *Lactobacillus brevis* CD2 were prepared. They show interesting anti-inflammatory properties due to their high levels of arginine deiminase [68]. The film is able to deliver lactobacilli inside the buccal cavity or towards the mucosa by just changing the application side, according to the desired local treatment, and proves their ability to be used in treating bacterial plaque in the oral cavity. The same polysaccharide, in combination with PVA, was used to obtain films by solvent casting and their physical–mechanical characteristics, morphology, mechanical properties, and disintegrating time, were evaluated. The successful incorporation of povidone-iodine and the ability of the system to deliver the drug consistently recommend it for applications in the treatment of periodontitis [69].

**Figure 3:**
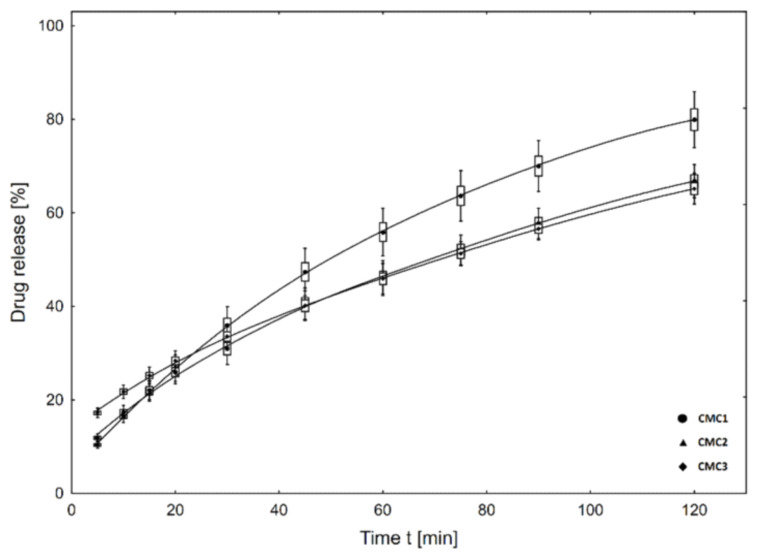
In vitro cumulative release profile of metronidazole from matrices based on CMCNa. Reprinted from ref. [70].

Metronidazole-loaded porous matrices based on gelatin and CMCNa or HEC were synthesized by whipping and lyophilization methods [70]. The matrices in the form of film were analyzed firstly from a morphological point of view and then swelling, degradation, and mechanical properties were also investigated. The applied matrices showed a high antibacterial efficacy against anaerobic *Bacteroides* sp. *Bacteria* and a moderate cytotoxicity in vitro on fibroblast and osteoblast cell cultures. The drug is released, in vitro, without any “burst effect”, the release efficacy varying between 60% and 80% after 120 min, depending on the composition of the film (Figure 3).

No adverse effects were showed by the metronidazole-loaded matrix based on hydroxyethyl cellulose after the clinical application. The decrease in periodontal pockets’ depth and bleeding was observed 1 month after a single application.

In conclusion of this section, the characteristics of polymer–drug systems in the form of films, mentioned above, are complemented by their mechanical properties that are superior to gels and hydrogels, which ensure a longer resistance at the site of application and, consequently, a longer period of the loaded drug release. Their disadvantage, compared to gels and hydrogels, is their lower drug loading efficiency, compensated by their release with a less pronounced “burst effect” but with a constant rate that ensures a constant concentration of the drug at the site of the disease, at the therapeutic level.

### 2.4. Fibers

Electrospun fibers can be produced from various polymeric materials, which can be biopolymers, synthetic, or a combination of both and have several practical applications. They can serve as drug delivery systems (DDS) or carriers of cells for tissue engineering. In this section, only the studies concerning the preparation of drug-loaded electrospun fibers, fabricated from biopolymers, for the treatment of periodontitis were taken into account. Generally, these fibers are placed in the periodontal pocket around the tooth with the help of an applicator and sometimes fixed with a cyanoacrylate adhesive [71]. The encapsulated drug, especially in the lumen fibers, is thus released directly into the periodontal pocket. A disadvantage, however, is that the introduction of fibers into the bag is a time-consuming operation, and some patients reported discomfort during their placement and a slight inflammation of the gums manifested by redness [72]. An example of such a system reports tetracycline-loaded collagen-based fibers, effective in treating chronic periodontitis for 3 months, which is even commercially available [73]. In another study, the authors obtained fibers from mixtures of alginate and glycerin cross-linked by ionotropic gelling (in the presence of Ba^2+^ cations) and loaded with ciprofloxacin and diclofenac sodium salt. This system has proven a good drug release and it is capable of inhibiting the growth of microbial cultures of *Escherichia coli*, *Escherichia fecalis*, and *Staphylococcus mutans* for over 10 days [74]. Other systems based on PCL were loaded with gentamicin sulfate. In this case, the system effectively suppressed the development of *Stapyhlococcus epidermidis* for 2 weeks [75]. 

Coaxial electrospinning was used for the preparation of electrospun fibers based on PLGA, gum tragacanth (GT), and tetracycline hydrochloride (TCH) as a hydrophilic model drug [76]. Drug release studies showed that both the fraction of GT and the core–shell structure can effectively control TCH release rate for 75 days with only 19% of burst release within the first 2 h. These membranes might be strong enough to be easily inserted into the periodontal pocket and sustainably release the incorporated drug, while affording patient compliance with low rigidity/stiffness of the membrane during the treatment. 

Two antibacterial agents, ampicillin (AMP) and metronidazole (MNZ), were loaded into a fiber mat system obtained from PLA [77]. This combination of drugs successfully suppressed *A. actinomycetemcomitans* in addition to the other pathogens, *F. nucleatum*, *P. gingivalis*, and *E. faecalis*.

Meloxicam (MX)-immobilized biodegradable CS-/PVA-/hydroxyapatite-based electrospun fibers were prepared by Yar et al., [78]. Smoother fibers were obtained at the highest drug concentration. 

A tinidazole (TNZ)-loaded CS/PCL mucoadhesive hybrid nanofiber membrane (TNZ-PCHNF) was prepared in order to alleviate the existing shortcomings in the treatment of periodontitis [79]. Antibacterial activity of this membrane (at TNZ concentration of 0%, 10%, 20%, and 30%, *w/w*) was tested against *S. aureus* (MTCC1303), and it appeared that the inhibition zone increased, as expected, with the increase in the drug concentration.

### 2.5. Microparticles

Such systems are solid polymeric particles, especially spherical in shape, with a diameter between 1 and 1000 μm and contain the drug dispersed throughout their volume. There may be physical, less often chemical (ionic bonds), interactions between the loaded bioactive agent and the constituent polymeric matrix, or the drug may only be adsorbed on the surface of the carrier. They are powdery materials obtained from both natural and synthetic polymers. The category of polymers usable for this purpose includes biodegradable ones, such as PLGA, polylactide, or poly(hydroxy alkanoates), or polysaccharides, such as CS, pectin, hyaluronic acid, etc. The following techniques are used to obtain microparticles: emulsification, solvent evaporation, coacervation, spray drying, electrospray, etc. [16]. The administration can be done in the form of suspensions, toothpaste, or by direct injection into the periodontal pocket [80]. The advantages of using these type of systems include the following: ensuring a controlled release, increasing patient compliance, and achieving a sustained therapeutic effect. 

Doxycycline-hiclate-loaded microparticles, having a sustained release for 47 days following a Fickian mechanism, have been shown to be effective in inhibiting microbial cultures of *Staphylococcus aureus*, *Porphyromonas gingivalis*, and *Staphylococcus mutans* [81]. Doxycycline hyclate and ornidazole were loaded in CS-vanillin cross-linked microspheres dispersed in situ gel (MLIG) implants [82]. In contrast to the previous study, the in vitro dissolution study demonstrated a non-Fickian type of drug release mechanism for 12 days. In addition, these formulations exhibited significant antimicrobial activity against *Staphylococcus aureus*, *Escherichia coli*, and *Enterococcus faecalis* and were found to be biocompatible and biodegradable during preclinical studies. Metronidazole-loaded CS particles, obtained by cross-linking in emulsion, showed a longer release compared to mixtures of the same microparticles and drug [83]. Microspheres of CS loaded with ornidazole (ORDZ) were prepared by the emulsification ionotropic gelation method [84]. These microspheres showed drug encapsulation in the range of 11.02 ± 0.98–32.45 ± 0.62% and sustained the release up to 5 days. The incorporation into a Pluronic (F-127) gel increased the release up to 7 days. The antimicrobial study indicated an inhibition of growth of *Staphylococcus aureus* at all drug concentrations. In another interesting study, microspheres, based on cationic CS and anionic xanthan gum, were prepared by coacervation and were further transformed into a gel that was loaded with chlorhexidine (CHX) [85]. CHX-containing gels exhibited selective antibacterial effects against the growth of *P. gingivalis*. CHX was also loaded into alginate-based microparticles and the authors have demonstrated that a higher amount of CHX was released from these microparticles in comparison with PLGA microparticles having the same particle size [86]. 

**Figure 4:**
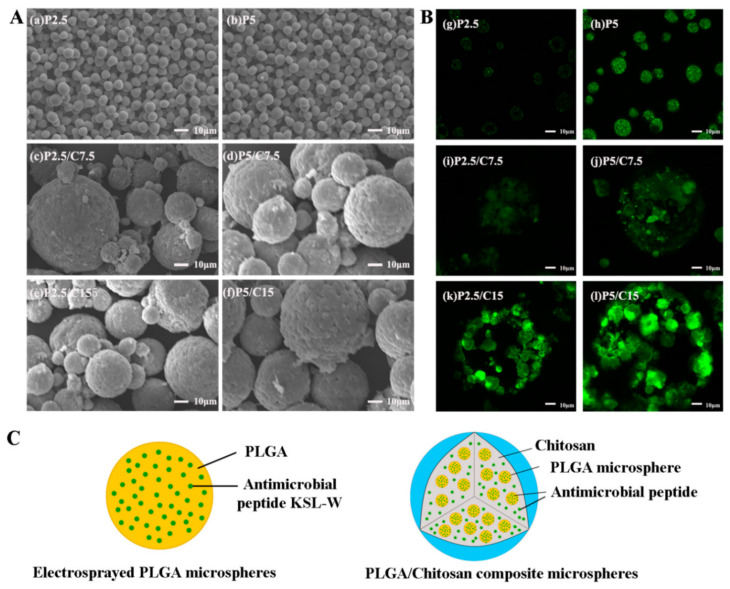
(**A**) Typical scanning electron microscope (SEM) morphologies of KSL-W-loaded PLGA and PLGA/CS composite microspheres; (**B**) visualized distributions of FITC-conjugated KSL-W in PLGA and PLGA/CS microspheres under laser scanning confocal microscope (LSCM); (**C**) a schematic diagram of a KSL-loaded PLGA microsphere and a KSL-loaded PLGA/CS microsphere. PLGA, poly(lactide-co-glycolide); CS, chitosan; FITC, fluorescein isothiocyanate. Reprinted from ref. [87].

An antimicrobial decapeptide, KSL-W (KKVVFWVKFK-CONH2), which could maintain stable antimicrobial activity in saliva was loaded into PLGA/CS composite microspheres (Figure 4), prepared by electrospraying and combined cross-linking–emulsion methods [87]. 

Antibacterial experiments demonstrated the prolonged antimicrobial and inhibitory effects of these KSL/PLGA/CS microspheres on oral bacteria. 

The combination of polysaccharides with bio-based polymers was also used in a study where interleukin 1 receptor antagonist (IL-1ra) was loaded into dextran/PLGA microspheres in order to evaluate the physicochemical characteristics and anti-inflammatory properties [88,89]. It was demonstrated that these microparticles blocked the IL-1β-induced production of pro-inflammatory cytokines in vitro.

Microparticulate polymer systems, according to experts in the field, have great potential for development in the near future as commercial products after performing several clinical tests.

### 2.6. Nanoparticles

Nanoparticles are the most promising strategy in the treatment of periodontal disease, because, due to their small diameter (between 10 and 1000 nm), they can penetrate regions that cannot be reached by other drug delivery systems. This advantage leads to a reduction in the frequency of administration and also ensures a more even distribution of the drug. Submicron polymeric particles with a diameter below 500 nm were prepared by many techniques, using in particular natural polymers (polysaccharides, proteins), biocompatible synthetic polymers, or mixtures thereof.

Table 2 briefly presents a series of results reported in the abundant literature of the last 3–4 years that illustrate different nanoparticle systems carrying drugs or other active principles with applicability in the treatment of periodontal disease.

CS is one of the most used polysaccharides for the preparation of nanoparticles, having the advantage of an intrinsic antibacterial activity [91]. It is often used in combination with alginates [100,101] or with PLGA [103,104,105], associated with the collagen matrix [97], in the form of derivatives [97,98] or biocomposites. 

Cellulose derivatives (CAP, HPMC, MC) [105,106] or other polysaccharides (sodium alginate) [102] are reported in recent literature. 

The brief presentation in this review highlights that polymer-based controlled release systems developed to date due to the benefits of nanotechnology are viable alternatives for the prevention and treatment of periodontal disease but require in-depth in vivo testing on animals and ultimately on human patients.

## 3. Conclusions

Periodontal disease is a worldwide health problem affecting people’s lifestyles. The best way to fight it is to prevent it, and the best way to do this is to remove its causes, particularly to prevent or remove the bacterial plaque. Classical methods of treating the disease are proving ineffective, which required finding a novel approach, with the potential use of nanotechnology being the most appropriate. The trend of recent years in the treatment of this disease is the local release of antibiotics, antimicrobials, and anti-inflammatory drugs, and the most appropriate method to achieve the desired effect is the local administration of drug delivery systems based on polymers. The advantages of this treatment are multiple because they directly target the affected area, maintain a relatively constant level of drug concentration, and minimize systemic side effects.

Formulations based on drug-loaded polymer systems are diverse, each with advantages and disadvantages.

Nanofiber-based films and membranes containing encapsulated drugs are easy to apply either on the gingival mucosa or by insertion into the periodontal pocket, but the amount of encapsulated drug is lower and its release faster. In addition, their removal must often be done by surgery, which reduces the patient’s compliance.

Gels and hydrogels administered by injection have the advantage of forming directly in the periodontal pocket, especially under the influence of external factors. The release of the drug is slower, especially from hydrogels, maintaining the constant concentration of the active principle for a longer time and thus increasing the effectiveness of treatment.

The advances registered by nanotechnology have allowed the realization of micro- and nanoparticle carrying drugs, which have a great potential for development in the near future as commercial products. This type of release system has been found to be the most viable alternative to date for the prevention and treatment of periodontal disease, but their implementation in current treatment requires in-depth in vivo testing on animals and ultimately on human patients.

## Figures and Tables

**Table 1:** Hydrogels based on polysaccharides with uses in the treatment of periodontal disease.

Cross-Linking Method	Polysaccharide	Cross-Linking Agent	Drug	Features/Administration Route	Biological Activity/Application	Ref.
Physical (ionic)	- CS	β-glycerophosphate disodium salt and gelatin	aspirin and erythropetin	- fast gelation induced by gelatin - drug release in vitro/in vivo—up to 21 days	- effective in anti-inflammation and periodontium regeneration	[36]
		β-glycerophosphate disodium salt	atorvastatin and lovastatin (nano-emulsions)	- thermosensitive hydrogel	-treatment of *Porphyromonas gingivalis* infected oral epithelial cells and gingival fibroblasts - decreased significantly pro-inflammatory markers expression (TNF-a and IL1ß) and pro-osteoclastic RANKL	[37]
		β-glycerophosphate disodium salt	dental pulp stem cell-derived exosomes		- suppressing periodontal inflammation - modulating the immune response	[38]
	- CS - quaternized CS	β-glycerophosphate disodium salt	chlorhexidine	- thermosensitive hydrogel - fast gelation at physiological temperature (6 min after insertion into the periodontal pocket) - release during 18 h	*Porphyromonas gingivalis*, *Prevotella intermedia*, *Actinobacillus actinomycetemcomitan*	[39]
	- carboxymethyl-hexanoyl CS	β-glycerophosphate disodium salt	naringin	- thermosensitive hydrogel - subgingivally administration	- therapeutic effect evidenced with micro-CT imaging, histology, the expression of inflammation-associated genes, myeloid differentiation primary response gene-88, and tumor necrosis factor-alpha	[40]
			N-phenacylthia-zolium bromide	- thermosensitive hydrogel - drug release in vivo—up to 30 days	- delay the initiation and facilitates the in vivo recovery from periodontitis	[41]
	- gellan gum - chlorhexidine - nanohydroxyapatite	ionic gelation with CaCl_2_	bone marrow mesenchymal stem	- injectable hydrogel	- inhibited *Enterococcus faecalis* - treating infectious bone defects caused by refractory periradicular periodontitis.	[42]
	- curdlan - polydopamine	ionic gelation	chlorhexidine acetate	- injectable hydrogel	- periodontal antibacterial treatment by combining photothermal effect and antimicrobial simultaneously - bacteriostatic rate until 99.9%.	[43]
Chemical (covalent)	- CS	GA	doxycycline hyclate	- fast sweeling in aqueous media with drug loading - release of 40 µg/mL drug after 24 h.	- periodontosis treatment and periodontal regeneration.	[44]
	- CS/hydroxyapatite	GA	recombinant human amelogenin	- mesoporous structure 7 nm in diameter of pores) - high surface area (33.95 m^2^/g) - high drug loading efficiency	- antibacterial effects against *Fusobacterium nucleatum* and *Porphyromonas gingivalis* - formation of bone and cementum-like tissue.	[45]
	- CS - PEO	GA	metronidazole glucose oxidase	- sensitivity to glucose (release of antimicrobial drug in response to the environmental glucose stimulus)	- drug release at higher glucose concentration - great capacity to inhibit *Porphyromonas gingivalis*	[46]
	- cellulose - cellulose nanofibers - κ-carrageenan oligosaccharide	- epichlorohydrin	surfactin and herbmedotcin	- 75% of the drug was released in vitro after 24 h.	- strong antibacterial activity against *Streptococcus mutans*, *Porphyromonas gingivalis*, *Fusobacterium nucleatum* and *Pseudomonas aeruginosa* - reduced the reactive oxygen species (ROS) generation, transcription factor, and cytokines production in human gingival fibroblast cells (HGF) under inflammatory conditions.	[47]
	- methyl cellulose	- xyloglucan from tamarind seeds	metronidazole	- high mucoadhesive property - superior injectability properties at 25 °C	- local drug carriers for periodontitis therapy	[48]
	- CS methacrylate	- photo-polymerization	metronidazole	- sensitivity to glucose (release of drug in response to the glucose stimulus)	- porous structure allows the control of the loaded metronidazole release with the glucose concentration rising. - local drug carriers for diabetics’ periodontitis therapy	[49]
double cross-linking (ionic and covalent)	- oxidized HA - collagen - oligomeric proanthocyanidins	- tricalcium phosphate - carbonylated hyaluronic acid	tetracycline	- macroporous morphology with interconnected pores whose diameter varies between 50 and 250 μm continuous release with an effectiveness of 93% after 5 days.	- local release of the drug into the periodontal pocket for the treatment of advanced chronic periodontitis	[50]
	- CS decorated metronidazole microcapsules included in a PVA matrix	4-carboxyphenyl-boronic acid	metronidazole	- bioadhesive - injectable directly in the periodontal pocket	- antibacterial effect for 14 days (in vitro tests) and for a week (in vivo) tests on a rat model of periodontitis	[51]

**Table 2:** Examples of nanoparticulate systems reported in the literature of recent years.

Polymer-Based Nanoparticles	Encapsulated Bioactive Compound	Preparation Method	Antibacterial Action Against	Application	Ref.
CS	Platelet-rich plasma	Ionic gelation method	*Staphylococcus mutans*	Complex/chronic wound healing and soft/hard tissue regeneration following periodontitis treatment or tooth extraction that needs prolonged growth factor release	[90]
	-	Precipitation from acetic acid solution with NaOH	*Enterococcus faecalis, Staphylococcus aureus, Bacillus subtilis*	Eliminating plasmid mediated resistance acquired by periodontal pathogens	[91]
	Amoxicillin and clavulanic acid	Ionotropic gelation with tri-polyphosphate	dentobacterial plaque	Higher efficacy by killing the pathogen bacteria in a sustained manner while reducing the cellular toxicity to non-bacterial cells	[92]
	Antimicrobial peptide	Ionotropic gelation with tri-polyphosphate and coating with the peptide	*Fusobacterium nucleatum, Porphyromonas gingivalis, Staphylococcus gordonii*	Treat root caries restorations to inhibit periodontitis related pathogens in periodontitis care	[93]
	Indocyanine green as a photosensitizer for antimicrobial photodynamic therapy	Ionotropic gelation with tripoly-phosphate	*Aggregatibacter Actinomycetemcomitans*	Potential implications for the treatment of *A. action-mycetemcomitans* infections in periodontitis and peri-implantitis in vivo	[94,95]
	Asiaticoside containing sulfobutyl ether-β-cyclodextrine complex	Ionotropic gelation		Carrier to deliver asiaticoside for periodontal tissue regeneration	[96]
CS-carboxymethyl CS	Doxycycline	Polyelectrolyte complexation and ionic gelation	*Porphyromonas gingivalis*	New option for the rational administration of doxycycline in the clinical treatment of periodontal disease	[97]
Lecitin-based liposomes coated with quaternary ammonium *N*,*N*,*N* trimethyl CS	Doxycycline	Electrostatic adsorption of CS derivative on liposome surface	*Porphyromonas gingivalis,* *Prevotella intermedia*	Potential applications in the clinical treatment of periodontal disease by extensive and efficient antibacterial activity	[98]
CS in collagen membrane	Chlorhexidine	Ionic gelation with sodium tri-polyphosphate	*Enterococcus faecalis*	Endodontic failure improves regenerative procedures in periapical surgery	[99]
Alginate coated CS core–shell nanoparticles	Transforming growth factor (TGF)-β1 and dexamethasone	Ionic gelation and polyelectrolyte complexation		Achieving healthy connective tissue ingrowth into the apical portion of the root canal space and subsequently a biologically based healing in root canal treatment	[100]
CS–sodium alginate polyelectrolyte complexes	Dimocarpus longan leaves extract	Polyelectrolyte complexation	*Staphylococcus aureus*	High antibacterial potential against bacteria that triggers periodontitis	[101]
Sodium alginate	Metronidazole	Emulsion–solvent evaporation method: single emulsion and double emulsification		Delivery of MNZ in periodontitis up to 24 h	[102]
CS coated poly(D,L-lactide-*co*-glycolide)	Lovastatin tetracycline	Double emulsion–solvent evaporation	*Aggregatibacter actinomycetemcomitans, Prevotella nigrescens*	Adjunctive treatment in periodontitis, promoted new bone formation in three-walled defects in beagle dogs	[103]
	Metronidazol *N*-phenacyl-thiazolium	Oil-in-water single emulsion–solvent evaporation		Reduced inflammation of experimental periodontitis, greater potential to resist further periodontal breakdown	[104]
	Simvastatin doxycycline	Double emulsion technology	*Porphyromonas gingivalis Staphylococcus sanguinis*	Loaded with SIM-DOX synergistically promoted the repair of the periodontium	[105]
Cellulose acetate phthalate	Chlorhexidine	Emulsion–solvent diffusion technique		Reduced the dentobacterial plaque index by 65.78%,	[106]
Hydroxyl propyl methyl cellulose, methyl cellulose, Carbopol 934	Coenzyme Q10	Nanoprecipitation, solvent evaporation, lyophilization		Management of chronic periodontitis	[107]

## Data Availability

Not applicable.
